# The Impact of COVID-19 Pandemic on University Faculty, Staff, and Students and Coping Strategies Used During the Lockdown in the United Arab Emirates

**DOI:** 10.3389/fpsyg.2021.682757

**Published:** 2021-09-09

**Authors:** Abdullah Seif Abdullah Al Miskry, Abdalla A. M. Hamid, Abdel Hameed M. Darweesh

**Affiliations:** ^1^Department of Cognitive Sciences, College of Humanities and Social Sciences, United Arab Emirates University, Al Ain, United Arab Emirates; ^2^Department of Clinical Psychology, College of Medicine and Health Sciences, United Arab Emirates University, Al Ain, United Arab Emirates

**Keywords:** COVID-19, university faculty members, university staff, students, distress, worry, coping

## Abstract

**Introduction:** The COVID-19 pandemic and the subsequent lockdown instigated serious mental health conditions. So far, the UAE data on mental health problems due to this pandemic outbreak is still scarce. The objective of this study was to identify the prevalent psychological difficulties experienced by university students, faculty members, and staff during COVID-19 lockdown and the coping strategies used.

**Methods:** A cross-sectional design was used to collect data from 737 participants using an online electronic survey. Participants included students, faculty members, and staff from universities in the UAE. The General Health Questionnaire (GHQ-12) was used to measure general distress, Penn State Worry Questionnaire (PSWQ-16) was used to measure worry, and the Coping Inventory for Stressful Situations (CISS-48) was administered to measure coping strategies used by participants during the COVID-19 pandemic lockdown. Data were collected during May to June 2020.

**Results:** The results indicated that 60.4% of students, 57.4% of the faculty members, and 52.3% of the staff experienced mild psychiatric problems. About 32.9% of students, 33.7% of the faculty members, and 25% of the staff experienced high levels of worry during the COVID-19 lockdown. Changes in eating patterns, worsening chronic health problems, change in sleep patterns, and concentration difficulties were reported. Furthermore, significant differences were observed in worry and coping strategies among participants. Women use more avoidance and emotion-focused coping compared to men.

**Conclusion:** It was concluded that COVID-19 lockdown has negatively impacted university faculty, staff, and students in terms of health behavior, psychological and physical health.

## Introduction

The new coronavirus (COVID-19) is a type of pneumonia first spotted in Wuhan, China (WHO, 2020a). The World Health Organization considers COVID-19 to be the sixth global public health concern (Guan et al., 2020). Its symptoms include cough, fever, muscle pain, sore throat, headache, loss of taste or smell, repeated shaking with chills, and difficulty breathing or shortness of breath (CDC, 2020). Clinical evidence indicates that older people and individuals with certain chronic illnesses such as lung disease, heart disease, and diabetes are at higher risk of getting infected with COVID-19 (CDC, 2020).

The COVID-19 pandemic has brought the greatest global challenge in this decade. The extent of the impact of this pandemic on global mental health and daily life is still mysterious. The unpredictable nature of the spread of this virus has brought great uncertainty within societies (Atchison et al., 2020; Verity et al., 2020), especially with the emergence of new variants of the virus (CDC, 2020). Researchers reported that about one-fifth of Iranians and almost a quarter of the Chinese population experienced severe to very severe levels of anxiety. Women were reported to experience more anxiety than men (Moghanibashi-Mansourieh, 2020; Wang et al., 2020).

Previous studies have shown increased distress associated with COVID-19 pandemic lockdown. For instance, some studies found that 22.8% of the participants experienced elevated stress (Yu et al., 2020). Furthermore, research revealed that two-thirds of the participants experienced psychological distress (Shahrour and Dardas, 2020). Moreover, another study demonstrated that about half of the participants experienced distress (Petzold et al., 2020).

In addition to the adverse effects of the disease, quarantining may also have a profound impact on mental health, such as fear of death, anger, and feeling of loneliness (Xiang et al., 2020). With more than 2.6 billion people living under some kind of quarantine, mental health cost is on the rise. The *Lancet* published a review of 24 studies documenting the distressing impact of quarantine on both public and healthcare workers. These impacts include depression, anxiety, anger, irritability, post-traumatic stress disorder (Brooks et al., 2020), distress, and worry (Kibbey et al., 2021). Other mental health problems incorporate low mood, insomnia, stress, and emotional exhaustion (World Economic Forum, 2020).

As mentioned above, worry is one of the major mental health issues related to the COVID-19 pandemic. Research has shown that worry is uniquely associated with anxious and depressive symptoms. Worry was found to be the dominant cognitive vulnerability factor that predicted increments in symptoms over time (Hong, 2007). One of the most stressful factors in worry is the unpredictability of the situation. In addition, the seriousness of the risk, and misinformation can heighten the sense of concern among the masses (Bao et al., 2020). Similarly, life challenges, such as the COVID-19 pandemic, and stress can trigger common mental health problems, such as anxiety, depression (Dar et al., 2017), and worry (Kibbey et al., 2021) that may need proper coping strategies to maintain individual mental well-being.

The worry induced by the COVID-19 pandemic is highly associated with psychological distress and may impact the coping strategies used by individuals (Rushabh, 2020). Regarding pandemic-related coping behavior, research has indicated that younger adults utilized a variety of coping strategies, such as avoidance and emotion-focused coping in an effort to control worry, compared to old adults (Hunt et al., 2003). In addition, age was found to be a significant factor in mental health as research has reported that COVID-19 pandemic quarantine affected people aged 21–40 years and above, in terms of their mental health condition (Ahmed et al., 2020). With regard to gender, research literature indicates significant gender differences in distress (Abu-Kaf et al., 2020; Bilodeau et al., 2020; Hamid and Abdullah, 2020; Olaseni et al., 2020), worry (Barahmand, 2008; Zlomke and Hahn, 2010; Bottesi et al., 2018; Domotor et al., 2019; Fu et al., 2020), and coping (Gemmell et al., 2016; Flannery et al., 2018; Martínez et al., 2019; Wang et al., 2020).

Literature reported that distress was associated with marital status during previous pandemic diseases (Babore et al., 2020). However, literature regarding the influence of marital status on distress during the outbreak of COVID-19 is inconsistent. Some studies argued that marital status was associated with distress-related insomnia and worry about family members; in contrast, others found no significant association (Fu et al., 2020; Li et al., 2020). For example, marital status was not a risk factor in psychological distress indicators, such as anxiety (Badahdah et al., 2020) and perceived distress (Babore et al., 2020).

Little is known about the psychological impact of COVID-19, and the ways faculty members, staff, and students use to cope with this quarantine in the UAE settings. However, research has uncovered that COVID-19 is possibly linked to worry (WHO, 2020b), anxiety (Kibbey et al., 2021), stress, and negative emotional reaction (CDC, 2020). Alcohol and other substances are also widely used by people in crisis to reduce negative emotions, distress, anxiety, or depression (Chodkiewicz et al., 2020). Therefore, this study explores the psychological impact of COVID-19 on university faculty members, staff, and students, and also the coping strategies used during the lockdown. The aim of this study was 3-fold: (1) to identify the prevalence of psychological difficulties experienced by faculty members, staff, and students during the COVID-19 lockdown; (2) to investigate the behavioral changes among participants during the COVID-19 pandemic lockdown; and (3) to identify the differences in worry, distress, and the coping strategies used during the COVID-19 lockdown with regard to gender, age groups, marital status, and categories of participants (faculty members, staff, and students). Based on the objectives of the study, the following hypotheses are stated: (1) psychological difficulties will be highly prevalent among faculty members, staff, and students during the COVID-19 lockdown; (2) participants will experience some behavioral changes during the COVID-19 pandemic lockdown; and (3) participant are expected to differ in worry, distress, and coping strategies with regard to demographic characteristics.

## Materials and Methods

### Sample

The sample is composed of 737 participants: 60.7% (*n* = 447) university students, 27.4% (*n* = 202) faculty members, and 11.9% (*n* = 88) staff selected through the convenience sampling method. The samples were selected from three universities (one public and two private) in Al Ain city, the emirate of Abu Dhabi, UAE. The common languages of the participants are Arabic and English. About 15.5% (*n* = 114) of the participants were aged 18 years and below, 39.9% (*n* = 249) aged 19–22 years, 5.3% (*n* = 39) aged 23–29 years, 8.1% (*n* = 60) aged 30–39 years, 16.8% (*n* = 124) aged 40–49 years, and 14.4% (*n* = 105) were 50 years and above. Around 72.6% (*n* = 535) of the participants were females, whereas 27.4% (*n* = 202) were males. Regarding marital status, 29% (*n* = 214) were married, and their families live with them in the UAE; 65.3% (*n* = 481) single, and 5.7% (*n* = 42) married, but their families live outside the UAE. For more description of the sample characteristics, see Table 1. The inclusion criteria for students in this study was to be enrolled during 2020/2021 academic year and for faculty and staff to be active employees in the universities. The participants with recent psychiatric diagnoses were excluded.

**Table 1:** Description of the demographic characteristics of the study sample.

**Variable**		**Students**		**Faculty**		**Staff**	
		***n***	**%**	***n***	**%**	***n***	**%**
Gender	Male	42	9.4	132	56.3	28	31.8
	Female	405	90.6	70	34.7	60	68.2
Marital status	Married	18	4	148	73.3	48	54.5
	Single	429	96	18	8.9	34	38.6
	Married (family is away)	0	0	36	17.8	6	6.8
Age Group	18<	114	25.5	0	0	0	0
	19–22	294	65.8	0	0	0	0
	23–29	39	8.7	0	0	0	0
	30–39	0	0	12	5.9	48	54.5
	40–49	0	0	92	45.5	32	36.4
	50>	0	0	98	48.5	8	9.1

*n = 737*.

### Measures

#### Demographic Information

Participants were requested to indicate their age, sex, marital status, and if they were faculty members, staff, or students. The participants were also asked to respond to the General Health Questionnaire (GHQ-12), the Penn State Worry Questionnaire (PSWQ), the Coping Inventory for Stressful Situations (CISS) and three other questions (worry about own health and health of their loved ones, behavior and health changes, and increased use of substances).

#### The General Health Questionnaire

The Arabic and English versions were used in the study. The GHQ-12 is composed of 12 items used to measure general distress (Goldberg and Williams, 1991). There are two methods of scoring the GHQ: one is the Likert-type scaling method (0, 1, 2, 3), which is used in survey research, and the other is the GHQ scoring method (0, 0, 1, 1), which is used to identify individuals with non-psychotic psychiatric disorders (Sallow et al., 2003). Both methods were used in this study, a cut-off of 6 was used with the GHQ scoring method to identify the percentages of non-psychotic psychiatric disorders (Endsley et al., 2017). The Arabic version was validated by Hamid and Musa (2010). The Cronbach's α reliability in the Arab sample was 0.94. The Cronbach's α in the current study was 0.81 (*M* = 18.22; SD = 5.25).

#### The Penn State Worry Questionnaire

The PSWQ is a self-report measure assessing clinically significant worry (Meyer et al., 1990). It consists of 16 items rated on a 5-point Likert-type scale, ranging from 1 = not at all typical of me to 5 = very typical of me (sample item: “I am always worrying about something”), depending on whether the item is worded positively or negatively. The cutoff point of 53 was used in this based on the literature on the Penn State Worry Questionnaire (Park et al., 2014). Adequate test–retest reliability of 0.74 was reported (VivWuthrich et al., 2014). The PSWQ was translated independently by three psychology professors following International Test Commission Guidelines for Translating and Adapting Tests (ITC, 2017), using a forward–backward translation method. The three professors are native Arabic speakers who completed their graduate studies in the Western universities. The Arabic version was given to a specialist in translation studies who translated it back into English to ensure semantic equivalence. In the current study, the Cronbach's α reliability was 0.85 (*M* = 48.26; SD = 8.13).

#### The Coping Inventory for Stressful Situations

The CISS comprises 48 items rated in a 5-point Likert type scale (Endler and Parker, 1994). Score 1 indicates not all engaged in the activity, and score 5 indicates very much engaged in the activity. The items are distributed in three major factors namely, task-focused, emotion-focused, and avoidance coping. Each factor consists of 16 items. Avoidance is further divided into two factors. These are social diversion coping and distraction coping (Cosway et al., 2000; Rafnsson et al., 2006). In the present study, avoidance was used as one factor. The Arabic version of this measure was already used in previous studies (Hamid and Abdullah, 2017; Hamid and Musa, 2017). The Cronbach's α reliabilities of CISS in two UAE samples were 0.74 (Hamid and Abdullah, 2017) and 0.88 (Hamid and Musa, 2017), respectively. In the current study, the Cronbach's α reliability of CISS is 0.86 (*M* = 158.54; SD = 21.71). With regard to the reliability of CISS dimensions, Choi et al. (2017) reported the alpha of 0.92 for task-focused, 0.88 for emotion-focused, and 0.86 for avoidance. For the current study, the Cronbach's α-values for task-focused coping was 0.86, emotion-focused coping was 0.84, and for avoidance was 0.82.

#### Procedure

A link of a survey composed of the online questionnaires and a section of demographic data (age, gender, categories, and marital status) was e-mailed to the participants after the Ethical approval from the Social Sciences Research Ethics Committee was granted (Ref No: ERS_2020_6114). The survey was e-mailed to participants during the COVID-19 lockdown from May to June 2020. The first page of the survey contained a consent form requesting the agreement of participants before responding to the questionnaire.

The objectives of the study and instructions on how the questionnaires would be responded to were clearly explained at the beginning of each questionnaire. They were informed of the voluntary nature of participation and the confidentiality policy. They were also informed that the data provided would only be used for research purposes and that their private information will never be revealed. Furthermore, they were also informed that they could withdraw from the study at any stage.

#### Data Analysis

The Statistical Package for Social Science (IBM SPSS, v26; IBM Corp., Armonk, NY, USA) was used to analyze the data. Skewness and kurtosis values were computed to test the normality of univariate distribution of the data. Skewness and kurtosis values were within the range of normality (±1.96) (Gravetter and Wallnau, 2014). Following the normality tests, descriptive analyses were performed to identify the levels of psychological difficulties and behavioral changes experienced by participants. The *t*-test and ANOVA were administered to examine group differences in distress, worry, and coping.

## Results

### The Prevalence of Mild Risk of Psychiatric Problems and Worry Among Faculty Members, Staff, and Students

Based on the cutoff point of 6, the results of the GHQ-12 indicated that 57.4% of the faculty members, 52.3% of the staff, and 60.4% of the students experienced mild risk of psychiatric problems. Regarding gender, 51.5% of the males and 61.3% of the females experienced mild risk of psychiatric problem. Concerning marital status, 57.7% of the married, 58.4% of the singles, and 66.7% of the married participants whose families are not in the UAE experienced substantial psychological difficulties.

With regard to worry, the results indicated that 33.7% of the faculty members experienced high level of worry during the COVID-19 lockdown compared to 25.0% of the staff and 32.9% of students as shown in Table 2. As for gender, 24.3% of the male participants and 35.1% the females experienced high levels of worry.

**Table 2:** The prevalence of mild risk of psychiatric problems and worry among faculty members, staff, and students during the COVID-19 pandemic.

**Variables**	**Category**					
	**Faculty members**		**Staff**		**Students**	
**GHQ-12**	***N***	***%***	***n***	***%***	***n***	***%***
No risk of psychiatric problems	86	42.6	42	47.7	177	39.6
Mild risk of psychiatric problems	116	57.4	46	52.3	270	60.4
**Penn-State Worry**						
Low worry	134	66.3	66	75.0	300	67.1
High worry	68	33.7	22	25.0	147	32.9

^*^Mild risk of psychiatric problems = Scored 6/12 on GHQ-12. χ2 (2, N = 737) = 2.17, p = 0.339.

^*^The cutoff point for worry is 53/80. χ2 (2, N = 737) = 2.39, p = 0.303.

Regarding marital status, 22.4% of the married, 33.9% of the single, and 61.9% of the married whose families are not in the UAE experienced high levels of worry (see Table 3).

**Table 3:** The prevalence of mild risk of psychiatric problems and worry across marital status during the COVID-19 pandemic.

**Variables**	**Marital status**					
	**Married**		**Single**		**Married but away** **from family**	
**GHQ-12**	***n***	**%**	***n***	**%**	***n***	**%**
No risk of psychiatric problems	91	42.5	200	41.6	14	33.3
Mild risk of psychiatric problems	123	57.5	281	58.4	28	66.7
**Penn State Worry Questionnaire**						
Low worry	166	77.6	318	66.1	16	38.1
High worry	48	22.4	163	33.9	26	61.9

^*^Mild risk of psychiatric problems = Scored 6/12 on GHQ-12. χ2 (2, N = 737) = 1.244, p = 0.537.

^*^The cutoff point for worry is 53/80. χ2 (2, N = 737) = 26.98, p = 0.00).

The researchers used three questions to measure health worry, behavior, and health changes of participants, and increased use of some substances. The first question measured worry about own health and worrying about the health of loved ones among participants. The results illustrated that about 18.5% of the participants reported worrying about their own health, whereas 81.5% reported worrying about the health of their loved ones (see Table 4). The majority reported worry about the health of their loved ones.

**Table 4:** Participants' responses to three questions measuring health worry, behavior, and health changes, and increased use of some substances.

**Questions**	***n***	**%**
**1. Worry about own health vs. worry about health of their loved ones**		
Own health	136	18.5
Health of loved ones	601	81.5
**2. Behavior and health changes**		
Change in eating patterns	66	22.5
Difficulty sleeping	144	19.5
Difficulty concentrating	133	18.5
Worsening chronic physical health problem	135	18.3
Worsening mental health	87	11.8
Other problems	72	9.8
**3. Increased use of substances**		
Alcohol	92	12.5
Tobacco	96	13
Coffee	189	25.6
Other drugs	65	8.8
No changes	136	18.9
Not applicable	156	22.2

The second question assessed about behavior and health changes of participants during the COVID-19 lockdown. The results indicated that 22.5% of the participants reported changes in eating patterns, 18.3% reported worsening chronic health problems, 19.5% experienced changes in sleep patterns, and 18% reported concentration difficulties. Furthermore, 11.8% of participants reported deterioration in mental health status. With regard to the third question, the results demonstrate that consumption of tobacco, alcohol, and coffee among participants increased during the COVID-19 pandemic lockdown (see Table 4).

### Gender Differences in Distress, Worry, and Coping

The result indicated significant gender differences in distress, worry, avoidance, and emotion-focused coping. Female participants consistently scored higher than males in these variables. There were no significant differences in task-focused coping (see Table 5).

**Table 5:** Gender differences in distress, worry, and coping.

**Variable**	**Male**		**Female**		***t***	***df***	***p***	**Cohen's *d***
	***M***	***SD***	***M***	***SD***				
Distress	5.41	3.31	5.93	2.2	−2.42	735	0.020	0.258
Worry	46.75	8.02	48.83	8.10	−3.12	735	0.002	0.185
Task	56.33	9.69	57.79	10.25	−1.76	735	0.08	0.146
Emotion	47.53	11.00	51.52	10.60	−4.51	735	0.000	0.369
Avoidance	49.11	12.12	51.34	10.32	−2.49	735	0.013	0.198

### Differences in Distress, Worry, and Coping Between Faculty Members, Staff, and Students

The ANOVA results showed significant differences in distress between faculty members, staff, and students [*F*_(2,734)_ = 5.471, *p* < 0.01, η^2^ = 0.02]. The *post hoc* results indicated that faculty members and students experienced more distress compared to staff (MD = 1.51, *p* < 0.05; MD = 2.00, *p* < 0.01, respectively). However, no significant differences were found in worry. As for coping, the results indicated significant differences in task-focused and emotion-focused coping [*F*_(2,736)_ = 3.564, *p* < 0.05, η^2^ = 0.010; *F*_(2,736)_ = 3.097, *p* < 0.05, η^2^ = 0.008, respectively]. There was no significant difference in avoidance coping.

### Differences in Distress, Worry, and Coping Across Marital Status

The ANOVA results showed significant differences in distress, worry, emotion-focused, and avoidance coping between single, married with families staying in the UAE, and those who are married but their families are outside the UAE. However, Eta-squared values suggest that these differences are small (see Table 6).

**Table 6:** ANOVA results of differences in distress, worry, and coping across marital status.

**Variables**		***n***	***M***	***SD***	***df***	***F***	***p***	**η** ^**2**^
Distress	Married (family in UAE)	214	5.79	3.59	2,736	6.526	0.002	0.017
	Single	481	5.67	1.85				
	Married (family is way)	42	7.14	2.95				
Worry	Married (family in UAE)	214	46.92	7.67	2,736	7.667	0.001	0.020
	Single	481	48.53	8.42				
	Married (family is away)	42	51.95	5.08				
Task	Married (family in UAE)	214	55.73	9.76	2,736	4.337	0.013	0.012
	Single	481	58.155	10.32				
	Married (family is away)	42	57.05	7.77				
Emotion	Married (family in UAE)	214	48.25	10.3	2,736	10.407	0.000	0.028
	Single	481	50.91	10.88				
	Married (family is away)	42	55.90	10.76				
Avoidance	Married (family in UAE)	214	50.48	10.98	2,736	6.634	0.001	0.018
	Single	481	50.33	10.80				
	Married (family is away)	42	56.62	9.76				

### Differences in Distress, Worry, and Coping Across Age Groups

The ANOVA results showed significant differences in distress and worry across age groups (see Table 7). With regard to distress, the Least Significant Difference (LSD) *post hoc* results showed that the younger group (18 and below) experienced more distress compared to the age groups of 19–22 and 40–49 years (MD = −1.70, *p* < 0.01; MD = −1.65, *p* < 0.05, respectively). Those aged 19–22 years old experienced less distress compared to the age groups of 30–39 and 50 and above (MD = 2.25, *p* < 0.01 and MD = 2.44, *p* < 0.001, respectively). The age group of 23–29 reported less distress than the age groups of 30–39 and 50 and above (MD = 2.31, *p* < 0.05 and MD = 2.49, *p* < 0.05, respectively).

**Table 7:** ANOVA results of distress, worry, and coping differences across age groups.

**Variable**	**Age group**	***n***	***M***	***SD***	***F***	***df***	**Sig**.	**η** ^**2**^
Distress	18 & below	114	17.32	2.78	5.923	5,731	0.000	0.039
	19–22	294	19.02	3.46				
	23–29	39	19.08	3.40				
	30–39	60	16.77	6.71				
	40–49	124	18.97	7.26				
	50 & above	106	16.58	7.41				
Worry	18 & below	114	49.50	7.23	2.475	5,731	0.031	0.017
	19–22	294	47.62	8.56				
	23–29	39	51.31	9.37				
	30–39	60	47.23	7.71				
	40–49	124	48.77	6.36				
	50 & above	106	47.53	9.03				
Task	18 & below	114	60.45	9.30	5.014	5,731	0.000	0.033
	19–22	294	57.46	10.70				
	23–29	39	56.69	10.21				
	30–39	60	54.47	8.93				
	40–49	124	54.98	8.94				
	50 & above	106	58.62	9.96				
Emotion	18 & below	114	52.39	10.50	7.402	5,731	0.000	0.048
	19–22	294	50.67	10.83				
	23–29	39	51.77	12.60				
	30–39	60	49.90	9.00				
	40–49	124	52.48	9.25				
	50 & above	106	45.02	11.63				
Avoidance	18 & below	114	51.95	10.72	5.519	5,731	0.000	0.036
	19–22	294	50.63	11.26				
	23–29	39	44.54	10.60				
	30–39	60	51.07	6.70				
	40–49	124	53.53	9.51				
	50 & above	106	48.51	12.27				

With regard to worry, ANOVA results showed significant group differences in worry across age groups (see Table 7). The LSD *post hoc* results showed that the age group of 18 and below reported less worry compared to the age group of 19–22 (MD = −1.99, *p* < 0.01). The age group of 19–22 reported more worry compared to those in the age group of 23–29 (MD = −3.69, *p* < 0.01), whereas the age group of 23–29 reported less worry compared to the age groups of 30–39 and 50 and above (MD = 4.07, *p* < 0.05 and MD = 3.78, *p* < 0.05, respectively).

The ANOVA results also showed significant differences between age groups in the use of task-focused, emotion-focused, and avoidance coping, [*F*_(5,731)_ = 5.014, *p* < 0.001, η^2^ = 0.033; *F*_(5,731)_ = 7.402, *p* < 0.001, η^2^ = 0.048; and *F*_(5,731)_ = 5.519, *p* < 0.001, η^2^ = 0.036, respectively] (see Table 7).

With regard to task-focused coping, the LSD *post hoc* results showed that the younger group (18 and below) used more task-oriented coping compared to the age groups of 19–22, 23–29, 30–39, and 40–49 years (MD = 2.99, *p* < 0.01; MD = 3.76, *p* < 0.05; MD = 5.98, *p* < 0.001; and MD = 5.46, *p* < 0.001, respectively).

About emotion-focused coping, the age group of 50 and above used less emotion-focused coping compared to the age groups of 18 and below, 19–22, 23–29, and 30–39 years (MD = 7.38, *p* < 0.001; MD = 5.66, *p* < 0.001; MD = 6.75, *p* < 0.01; and MD = 4.88, *p* < 0.01, respectively).

As for avoidance coping, the results indicated that the younger age groups used more avoidance coping compared to the older groups. The age group of 18 and below used more avoidance coping compared to the age groups of 23–29 years (MD = 7.74, *p* < 0.001) and the age group of 50 and above (MD = 3.44, *p* < 0.01). The age group of 23–29 used more avoidance coping compared to the age groups of 30–39 (MD = 6.53, *p* < 0.01), and the age group of 40–49 used more avoidance coping than the age group of 50 and above (MD = 5.02, *p* < 0.001).

## Discussion

The findings of the study suggest that about 57.7% of the faculty members, 52.3% of the staff, and 32.9% of the students scored 6/12 or more on the GHQ-12. Thus, the first hypothesis, which posited that psychological difficulties would be highly prevalent among participants during the COVID-19 lockdown, is supported. This result indicates a high prevalence of mild risk of psychiatric problems among participants during the COVID-19 lockdown. These findings are consistent with Petzold et al. (2020) findings that found over 50% of the participants expressing elevated levels of psychological distress related to the COVID-19 pandemic. Similarly, Son et al. (2020) found that 71% of students reported heightened stress and anxiety related to the COVID-19 pandemic. Furthermore, Wang et al. (2020) reported moderate-to-severe psychological difficulties among the general population in China during the initial stage of the COVID-19 pandemic.

The high prevalence of psychological difficulties in the present study was further supported by the responses of participants to a question on mental health status in which over 11.8% of them perceived deterioration in their mental health status. This result is supported by the findings of Lyons et al. (2020) who reported a high percentage of mental well-being deterioration among Australian students during the COVID-19 pandemic.

However, the findings of the current study suggest that participants may not be fully aware of the real impact of COVID-19 lockdown on their mental health as their response to the direct question about the deterioration of mental health was not consistent with the results of the GHQ-12. Nonetheless, more than 18% of the participants reported worsening chronic physical health.

The age group of 19–22 experienced lower levels of distress compared to the other age groups except for the age group of 18 and below. The youngest group (18 and below) experienced a higher level of distress that could be due to being in their first year at the University where they had to deal with both the challenges of being junior students and the demands of COVID-19 lockdown. These findings are in line with previous studies by Shahrour and Dardas (2020) and Alkhamees et al. (2020).

With regard to worry, the findings suggest that more than 33% of the faculty members, 25% of staff, and 32.9% of students experienced a high level of worry during the pandemic lockdown. They were more worried about the health of their loved ones (81.5%) than about their own health (18.5%). This result is consistent with the previous studies that found high levels of fear and worry among individuals about the health of their loved ones compared to their own health (Son et al., 2020).

The second hypothesis postulated that participants would experience some behavioral changes during the COVID-19 pandemic lockdown. The findings suggest that the most affected behaviors were coffee consumption, eating patterns, sleeping difficulties, concentration difficulties, increased use of tobacco, and alcohol consumption. Hence, the second hypothesis of the study is supported. These findings are consistent with the previous studies that reported a higher percentage of concentration difficulties and disruptions in sleeping patterns among students (e.g., Son et al., 2020). The findings are also consistent with a previous study, which reported 14% increase in alcohol consumption during COVID-19 “Lockdown” in Poland (Chodkiewicz et al., 2020). Likewise, Czeisler et al. (2020) reported that 13.3% of the participants experienced increased substance use during COVID-19 lockdown. However, only 18.9% of the participants in our study reported no changes in their behavior.

The third hypothesis suggests that participants would differ in worry, distress, and coping strategies with regard to demographic characteristics. The results supported this hypothesis. It is clear from the findings that faculty members and students experienced greater levels of distress compared to staff. This may be due to the demanding nature of online teaching and the lack of face-to-face interaction.

The findings suggest that women use more avoidance and emotion-focused coping during COVID-19 lockdown than do men. This indicates that men may be more capable of adapting to the demands of the COVID-19 lockdown contrary to what was suggested by previous research (Umucu and Lee, 2020). Consistent with the previous studies (Abu-Kaf et al., 2020; Bilodeau et al., 2020), we found that women experience more distress than did men. The greater level of worry experienced by women in this study compared to men is similar to that found by Bottesi et al. (2018) and Domotor et al. (2019). This result is inconsistent with the findings of Zlomke and Hahn (2010) where men were found to experience more worry compared to women.

With regard to marital status, the findings denoted that the single participants used more task-focused coping compared to the married ones. Those who are married but their families are outside the UAE reported more distress, worry, and avoidance coping compared to singles and married whose families are in the UAE. These results are inconsistent with the findings that unmarried individuals were more likely to experience heightened distress compared to the married participants (Yu et al., 2020).

In terms of age groups, those aged 22–29 reported more distress and worry compared to the other age groups. The older group appeared to use more emotion-focused and avoidance coping in dealing with distress and worry related to the COVID-19 lockdown, whereas the younger age group (18 and below) seemed to use more task-focused coping.

## Conclusion

The COVID-19 lockdown has negatively impacted the psychological and physical health of faculty members, staff, and university students. More than 57% of faculty members, 52% of staff, and 60% of students experienced mild risk of psychiatric problems. Females seem to be more susceptible to these problems. Special attention needs to be directed toward married individuals whose families are not living with them during the pandemic lockdown as they are most prone to mental health. Online counseling might be useful to help them deal with the psychological distress they experience. In addition, equipping them with effective coping skills may enhance their resilience in such situations. Furthermore, more reliable and up-to-date information about the COVID-19 prevention could reduce the fear and distress they experience. The COVID-19 lockdown increased the use of the substance, such as tobacco, alcohol, and coffee. Females seem to use more avoidance and emotion-focused coping in dealing with the demands of COVID-19 lockdown. Those aged 40–49 also seem to use more emotion-focused and avoidance coping. In addition, participants seemed to worry more about the health of their loved ones than about their own health. This population may be resurveyed at the end of the pandemic lockdown to examine the long-term psychological impact of COVID-19 among the university communities. Overall, the COVID-19 has posed a very high demand, especially on the faculty members and university students. This is evident in the elevated psychological difficulties such as worry and distress that necessitate behavioral changes aimed at managing this situation.

## Limitations

One limitation of the current study is the use of an online survey in data collection. Online surveys are associated with low response rates that may negatively affect the generalizability of findings (Sivo et al., 2006; Mulvany et al., 2019). However, this method was the only available means to collect data from participants during the COVID-19 lockdown. Further, the current study is exploratory and cross-sectional in nature. Hence, advanced designs may be appropriate to explore causal associations among the study variables. Furthermore, a convenient sampling method was used in this study, which may not be appropriate to draw a representative sample of the population. Therefore, future studies may use more fine-grained analysis to obtain more comprehensive results.

## Data Availability Statement

The original contributions presented in the study are included in the article/supplementary material, further inquiries can be directed to the corresponding author/s.

## Ethics Statement

The studies involving human participants were reviewed and approved by Social Sciences Research Ethics Committee, United Arab Emirates University. The patients/participants provided their written informed consent to participate in this study.

## Author Contributions

All authors contributed to the manuscript planning, data collection, the preparation of this conceptualization, data curation, formal analysis, investigation, methodology, project administration, resources, validation, writing the original draft, writing, review, and editing.

## Conflict of Interest

The authors declare that the research was conducted in the absence of any commercial or financial relationships that could be construed as a potential conflict of interest.

## Publisher's Note

All claims expressed in this article are solely those of the authors and do not necessarily represent those of their affiliated organizations, or those of the publisher, the editors and the reviewers. Any product that may be evaluated in this article, or claim that may be made by its manufacturer, is not guaranteed or endorsed by the publisher.
